# Tyranny over Women

**Published:** 1875-08

**Authors:** 


### THE TYRANNY OVER WOMAN.

There are about twelve hundred millions of human beings on this globe of ours, and of these, probably not far from one-tenth permit the most terrible tyranny to be exercised over its women. The female portion of the other nine-tenths have their particular griefs and hardships and endurances, but their tortures are light as compared with the sufferings of the one-tenth.

The one-tenth are the civilized, refined, polite peoples of the earth ; the nine-tenths are the semi-civilized and barbaric. The tyranny is the tyranny of fashion, the despotism of public sentiment. The leading tyrant is some person whom nobody knows ; for whose private and personal opinion no mother in the land would care two straws, and whom perhaps she would no more invite to her home than she would a pestilence. Yet the tyranny is submitted to by all, and nobody who is anybody, dares remonstrate or rebel. The tyranny is not only quietly submitted to, but there is always an actual strife to determine who among the enslaved shall be the first to wear the fetters, newly forged from season to season. It is bad enough when this tyranny merely extends to matters of taste ; it is fearful when it affects morals and health. That it has tended to impair the former in times past will be asserted by those who do not favor decollette dresses ; that health suffers to a greater or less extent through this tyranny is undeniable. We have but to point to the multitudes of waists injuriously compressed by corsets ; to the permanent suffering brought about by piles of false hair and the application of poisonous dyes and cosmetics ; to the local discomfort and internal derangement induced by distorting shoes and high-heels, in order to satisfy the most careless observer that the despotism of which we speak has done its full share in the way of dethroning the health of its victims. The supremacy of dress has gradually become a toil and torment. To be well dressed for a woman now-a-days, is to sell herself, soul as well as body, to the omnipotence of clothes. For garments have ceased to be a mere covering. They are to the wearer as the petals are to a flower, as the rind is to a fruit, as the foliage is to a tree, as the clouds are to a sunset. A woman's charms are to-day one-third her own, and the other two-thirds are owing to her dressmaker, milliner, and hair-dresser.

Who shall deliver the fair sex from this tyranny ? Who shall bring back the simple styles of other days, the plain skirts, the plainer hair, the general symplicity of attire that once was prevalent ?

Fashion is at best but a capricious goddess. Worse still, she is an inexorable one. One season she piles a lady's tresses mountains high above her forehead. The next, she sends them to dangle on a pigtail down the wearer's back. In the spring she loops a lady's skirt above her boot-top. In the autumn she bids her victim trail yards of silk behind her

Ordinarily fashion appears to set all chronology at defiance in the matter of woman's costume. She demands the "pompadour" in hair, the Elizabethan in ruffles, the Louis XV. in high-heels, and the nondescript in tight skirts. She is called a goddesss ; she is certainly as cruel as inconsistent. Time was when she demanded that every woman should court death by walking the streets in thin-soled slippers. Now they defy the weather in stout and comfortable boots. But there is no certainty that the thin slipper may not come into vogue again. And if it does, hundreds of fashionable ladies will, on account of it, cough themselves into untimely graves.

---

Happiness and joy are the inevitable consequences of virtuous actions, and these are the only substantial benefits of life. Ill-gotten pleasures are void of that vitality which is necessary [illegible] make them lasting.

